# Editorial: Implementation of Evidence-Based Digital Health Interventions to Support Public Health

**DOI:** 10.3389/fpubh.2022.913150

**Published:** 2022-05-09

**Authors:** Hajo Zeeb, Julian Wienert, Tina Jahnel, Rehana Shrestha

**Affiliations:** ^1^Department of Prevention and Evaluation, Leibniz Institute for Prevention Research and Epidemiology (LG), Bremen, Germany; ^2^Faculty of Human and Health Sciences, University of Bremen, Bremen, Germany; ^3^Department for Health Services Research, Institute of Public Health and Nursing Research (IPP), IU Internationale Hochschule, Bremen, Germany; ^4^Department of Social Epidemiology, Institute of Public Health and Nursing Research (IPP), Bremen, Germany

**Keywords:** digital health, implementation, public health, evidence-based, technology

With the Corona pandemic, many health systems have experienced a substantial increase in the use of digital technologies both in terms of patient care and public health. One notable example are the manifold versions of Corona Apps providing risk information and indicating contact and vaccination status, among others. These apps may have had public health surveillance functions, but often also served as information intervention to support protective behavior such as taking a test once a warning appeared. Video consultations became widely used in many countries as patients were advised or forced to stay at home and avoid contacts. These are only selected examples of recently implemented digital interventions. While they supported essential public health functions, they also provided numerous stories of success and of failure. Moreover, while aiding the functioning of health care and prevention in challenging times, some of the digital solutions put additional stress on an already burdened health workforce. This broad array of issues related to implementation of digital health interventions–though unrelated to the Corona pandemic when initially conceived–was at the core of the current selection of research contributions combined under this Research Topic.

While a digital, i.e., communication-technology based approach is the unifying component of the interventions in this topic, some conceptual differences should be noted. Whereas ehealth generally encompasses the use of information and communication technologies to support all aspects related to health and health services (included health education and health research), mhealth as a component of ehealth describes medical and public health activities that are supported by mobile devices such as smartphones, tablets etc. There is no clear delineation of digital health against these two definitions, however, digital health may be seen as including ehealth (and mhealth) and telemedicine as well as health technology such as wearable sensors and trackers. Digital health is a very dynamic field with a focus on emerging uses of information and communication technologies, including artificial intelligence for health. Contributions in the current Research Topic address various aspects of the increasingly broad scope of digital health that also permeates public health.

The paper by Wienert and Zeeb uses an important theoretical implementation science model—the Consolidated Framework for Implementation Research. The framework draws upon constructs from several other implementation theories, thus providing an overarching typology supporting the examination of what works, where and why. Considering it as a valuable instrument that might provide formative evaluations for the implementation and monitoring of health information technology, the authors demonstrate its adaptation to identify elements for a successful implementation of health apps. Their paper provides a first step toward developing an implementation framework while stressing the need to include digital concepts within such framework to monitor digital health apps at large.

Patients with chronic diseases were at particular risk during the Corona pandemic, not only because the Corona infection could lead to more severe disease but also because usual healthcare was at risk of being interrupted or delayed. Seixas et al. provide a nuanced overview of telemedicine solutions for chronic disease management, highlighting positive and negative aspects of telemedicine for this purpose. Their paper describes specific steps to better implement digital health solutions for patients with chronic diseases, stressing interdisciplinary team approaches and user engagement.

For the large and growing group of patients with hearing loss, Murdin et al. assess hearing impaired patients' acceptance and usability of hearing support system connected to a mobile smartphone app. Their paper highlights that both older and younger adults are generally satisfied with the hearing aid connected to a mobile smartphone app that allows remote control of their hearing aids, provides instructional videos, audiological tests and auditory training. Nonetheless, the authors suggest minimization of technical problems related to Bluetooth connectivity, availability of easy-to-use personalized guidance on monitoring tests and auditory training on a mobile phone as critical to promoting self-management and improving users' satisfaction and uptake of such technology.

Finally, in another paper looking at specific problems of restricted social support services caused by the Corona pandemic, Hoel et al. explored the impact of COVID-19 on community-dwelling dyads in a dementia caregiving context and the feasibility of social technology to promote and support social participation and dyadic relationship. Using a qualitative study design, the authors shed light on an interesting intertwining of dyads' prior (in)active social life before the pandemic and their familiarity with social technology. While the socially active dyads familiar with social technology were able to better cope with pandemic-related restrictions, dyads who were socially active prior to pandemic but with limited use of technology found the situation stressful due to disconnections with their social contacts. Furthermore, the paper describes that barriers to using digital technology among older adults are not due to lack of willingness among this group but rather insufficient support and tech literacy, thus stressing the need to customize such technology to the individuals' needs and conditions.

Digital technology moves fast, and careful implementation research with a view to understanding both the benefits and risks associated with digital technology is of great and growing importance. The papers in this Research Topic contribute to this discussion. We hope that readers find them useful to inspire their own future work.

## Author Contributions

HZ and RS wrote the first draft. All authors read and revised the draft, contributed to the final version, and approve of the final version.

## Funding

This work was supported by the Leibniz Science Campus Digital Public Health Bremen.

## Conflict of Interest

The authors declare that the research was conducted in the absence of any commercial or financial relationships that could be construed as a potential conflict of interest.

## Publisher's Note

All claims expressed in this article are solely those of the authors and do not necessarily represent those of their affiliated organizations, or those of the publisher, the editors and the reviewers. Any product that may be evaluated in this article, or claim that may be made by its manufacturer, is not guaranteed or endorsed by the publisher.

